# Case Report: The first familial hCG syndrome in a Chinese family

**DOI:** 10.12688/f1000research.53636.1

**Published:** 2021-06-08

**Authors:** Ling-Yin Hung, Mei-Tik Leung, Toby Chun-Hei Chan, Hoi-Ning Cheung, Wai-Hon Li, Yui-Shing Cheung, Assumpta Sze-Man Wong, Chi-Chung Shek, Sammy Pak-Lam Chen

**Affiliations:** 1 Chemical Pathology Laboratory, Department of Pathology, Queen Elizabeth Hospital, Kowloon, Hong Kong; 2 Chemical Pathology Laboratory, Department of Pathology, Princess Margaret Hospital, Hong Kong; 3 Department of Obstetrics & Gynaecology, Queen Elizabeth Hospital, Kowloon, Hong Kong

**Keywords:** familial hCG syndrome, hCG, human chorionic gonadotropin, gestational trophoblastic disease, ectopic pregnancy, tumour marker, autosomal inheritance

## Abstract

Familial hCG syndrome is a rare and benign cause of elevated serum beta human chorionic gonadotropin (hCG). We present here a case of familial hCG syndrome diagnosed in a Hong Kong Chinese family, which we believe to be the first reported in Chinese. A 38-year-old woman presented with incidental finding of persistently elevated hCG, analytically confirmed both in urine and blood. Extensive radiological and biochemical work-up were performed but were negative for pregnancy and malignancy. Testing of another asymptomatic family member revealed unexplained elevation of serum hCG, confirming the diagnosis of familial hCG syndrome. Knowledge and awareness of this entity among clinicians are important to avoid unnecessary investigations and treatment in affected families.

## Introduction

Familial hCG syndrome is a rare but benign cause of elevated serum/urine hCG. To date, fewer than 20 families have been reported worldwide in the literature ^1– 3^. Patients present with an otherwise unexplained elevation in serum/urine hCG, and diagnosis is established by exclusion of other causes of elevated hCG and testing of family members. An autosomal dominant pattern of inheritance was observed, although the molecular mechanism has been poorly defined. We present here a case of familial hCG syndrome diagnosed in Hong Kong, which we believe to be the first reported family in Chinese.

## Case presentation

A 38-year-old Chinese woman, a veterinary surgeon, was referred for investigation of persistent unexplained elevation in serum beta human chorionic gonadotropin (hCG) levels first revealed in a positive urine pregnancy test for the investigation of abdominal pain in August 2020. Apart from a history of menorrhagia and iron deficiency anaemia, the patient had unremarkable past health. She was not on any drugs or supplement and had never been sexually active. Family history was unremarkable. Blood tests showed stable elevation of total beta-hCG ranging from 93–112 IU/L by Roche Elecsys HCG+β assay over the subsequent five months. Transabdominal ultrasound of the pelvis showed no intrauterine sac, and no abnormality was detected in the uterus and ovaries. Pituitary hormones, including serum thyroid-stimulating hormone (TSH), luteinizing hormone (LH), follicle stimulating hormone (FSH) and prolactin levels were normal. Biochemical findings of the patient are summarized in Table 1.

**Table 1.** Summary of biochemical results of the patient.

Assay	Result	Reference intervals
Serum results		
hCG		
Roche Elecsys hCG+β	98 IU/L	≤6 IU/L
Abbott Architect Total β-hCG	30 IU/L	<5 IU/L
Siemens Immulite 2000 total β-hCG	424 IU/L	<5 IU/L
Beckman DxI total β-hCG	37 IU/L	<5 IU/L
Hyperglycosylated hCG (hCG-H)	<0.5 μg/L	<1.0 μg/L
LH	3.6 IU/L	Follicular 2.4 – 12.6 Ovulation 14.0 – 95.6 Luteal 7.7 – 58.5
FSH	2.1 IU/L	Follicular 3.5 – 12.5 Ovulation 4.7 – 21.5 Luteal 1.7 – 7.7
Prolactin	251 mIU/L	102 – 496 mIU/L
TSH	3.08 mIU/L	0.27 – 4.20 mIU/L
CEA	<1.8 μg/L	≤4.7 μg/L
CA125	11 U/mL	≤35 U/mL
AFP	<2 IU/mL	≤6 IU/mL
Urine results		
Roche Elecsys hCG+β	168 IU/L	NA

hCG: human chorionic gonoadotropin LH: Luteinizing hormone FSH: Follicle stimulating hormone TSH: Thyroid stimulating hormone CEA: Carcinoembryonic antigen CA125: Cancer antigen 125 AFP: Alpha fetoprotein

 Due to the discordant clinical and biochemical findings, analytical interference of hCG assay was suspected and a series of interference studies were performed ( Table 2). Serial dilution study was performed on the Roche Elecsys HCG+β assay with manufacturer diluent in 1:4 and 1:9 dilutions, which showed linear recovery. Satisfactory recovery was observed in serum hCG level on the Roche assay after treatment with heterophilic blocking tube (Scantibodies Laboratory, Inc). Polyethylene glycol (PEG) precipitation study was performed by mixing 200μL of patient sample with 200μL of 25% (w/v) PEG 6000 (Sigma Aldrich). The sample was then incubated at room temperature for 10 minutes and centrifuged at 9500 × g for 10 minutes. hCG levels were assayed by the Roche Elecsys HCG+β assay in the supernatant. Recovery was satisfactory compared to the hCG result in the neat untreated serum sample. Aliquots of serum were sent to three local laboratories for analyses using different analytical platforms (Abbott Architect, Beckman DxI and Siemens Immulite 2000), all confirming elevation in beta-hCG but to variable extents, with the highest hCG result obtained using Siemens Immulite 2000 ( Table 1). Spot urine total beta-hCG was checked by the Roche Elecsys HCG+β assay and measured 168 IU/L.

**Table 2.** Results of interference studies performed on patient’s serum specimen.

Dilution recovery	hCG in neat sample: 112 IU/L hCG with x5 dilution: 106 IU/L (Recovery: 95%) hCG with x10 dilution: 103 IU/L (Recovery: 92%)
Heterophilic blocking tube	hCG in neat sample: 112 IU/L hCG post blocking tube treatment: 107 IU/L (Recovery: 96%)
PEG precipitation	hCG in neat sample: 105 IU/L hCG in supernatant after 1:1 PEG treatment: 100 IU/L (Recovery: 95%)

hCG: human chorionic gonoadotropin PEG: Polyethylene glycol (PEG 6000)

To look for underlying tumours, tumour markers including carcinoembryonic antigen, cancer antigen 125 and alpha fetoprotein were checked but were not elevated. Hyperglycosylated hCG (hCG-H) was undetectable (<0.5 μg/L) using the Nichols Advantage hCG-H assay (Nichols Institute Diagnostics, San Clemente, California). Whole body positron emission tomography-computed tomography, magnetic resonance imaging of the pituitary, abdomen and pelvis were unrevealing. Oesophagogastroduodenoscopy was negative.

 As biochemical and radiological work-up could not explain the persistently elevated hCG, familial hCG syndrome was considered in this otherwise asymptomatic patient. Blood hCG test was arranged for one female sibling who also had never been sexually active, and the level was elevated (37 IU/L). There were no other family members available for testing. The diagnosis of familial hCG syndrome was thus established. This is to the best of our knowledge the first case of familial hCG syndrome reported in Chinese.

## Discussion

 Human chorionic gonadotropin is a heterodimeric glycoprotein hormone produced by placental syncytiotrophoblast during pregnancy ^4^. It comprises of a common alpha subunit shared between LH, FSH and TSH, and a unique beta subunit that has been shown to be encoded by at least seven genes ^4, 5^. In blood circulation, hCG exists in various forms arising from post-translational glycosylation and its degradation in the liver and kidneys ^4^. Apart from pregnancy, increased levels may be seen in gestational trophoblastic diseases, and a number of tumours including germ cell tumours, non-small cell lung cancer ^6^, as well as squamous cell carcinoma of the head and neck region ^7, 8^, in which other hCG forms such as free beta-subunit or hyperglycosylated form may predominate ^4^. In postmenopausal women or patients with gonadal insufficiency, sulfated hCG secreted from the pituitary is a frequent cause of mildly elevated hCG. Occasionally, elevated hCG may arise from exogenous administration in doping athletes or as a dietary supplement. In a patient who has otherwise unexplained elevation of hCG discordant with clinical findings, analytical interferences, such as heterophilic antibody interference, are important causes that should be excluded. According to data from USA, hCG Reference Service, among 424 cases of positive hCG with no history of gestational trophoblastic disease or malignancy and pregnancy excluded, the commonest aetiologies were quiescent gestational trophoblastic disease (32%), analytical interference (25%) and pituitary hCG (23%), followed by other malignant conditions (15%) and exogenous intake (1%) ^1^. 

The above differentials were, however, unlikely in our patient based on the extensive negative biochemical and radiological work-up. Intrauterine and extrauterine pregnancies were unlikely given the negative pelvic imaging. Active gestational trophoblastic disease and occult malignancies were less likely with the negative findings on extensive whole-body imaging and undetectable hyperglycosylated hCG. The normal LH and FSH ruled out pituitary hCG. Analytical interferences were less likely although not entirely excluded by the normal recovery on serial dilution, treatment with heterophilic antibody blocking tube and PEG precipitation. The detection of hCG in the urine provides further evidence for a genuine increase in endogenous hCG levels. The negative work-up and persistent elevation in hCG led to the suspicion of familial hCG syndrome, which was subsequently confirmed with testing of hCG level in an asymptomatic family member.

Familial hCG syndrome is a rare but benign cause of elevated hCG. Serum hCG from reported cases in literature ranged from <1.0 – 216 IU/L, and levels showed fluctuation with time on serial testing ^2^. Antibody profiling by Cole demonstrated the predominance of free β-subunit and β-subunit missing the C-terminal peptide in affected patients ^1^. Both forms are biologically inactive, thus explaining the unaffected fertility in these patients. The presence of these uncommon hCG variants may account for the difference in hCG results across different analytical platforms. Studies comparing the reactivity of available international standards and international reference preparations with sandwich immunoassays on major analyzers have shown vastly different recoveries towards different hCG variants ^9– 11^. For instance, the Siemens Immulite 2000 total β-hCG assay, which was shown to have equimolar detection of β-subunit missing C-terminal peptide and nicked hCGβ, consistently returned the highest hCG result among the four platforms tested in our patient. The incongruent results across multiple immunoassay platforms may provide additional support to the diagnosis of familial hCG syndrome, as direct measurements of the various hCG subtypes are not readily available in routine clinical practice. Knowledge of the assay reactivity towards the different variants may aid in the interpretation of discordant results.

Reliable markers or diagnostic tests specific to familial hCG syndrome are lacking. Diagnosis relies on careful exclusion of common causes and testing of family members, the former often includes performing a number of costly diagnostic imaging. Of note, a lack of response to a trial of methotrexate therapy has been proposed as one of the diagnostic criteria for familial hCG syndrome in female patients of reproductive age to exclude ectopic pregnancy ^1^, which, however, is not without risk. Further research into the underlying molecular mechanisms and structural characterization of the variants may identify better means of establishing a diagnosis, and may allow detection of concurrent physiological or pathological hCG elevations in diagnosed patients.

In conclusion, we report here the first case of familial hCG syndrome in a Chinese family. Given the rarity of the entity and the lack of readily available biochemical markers, familial hCG syndrome remains a diagnosis by exclusion. Knowledge and awareness of this disorder among clinicians are important to avoid over investigations and unnecessary treatment, and in offering reassurance to affected patients and family members.

## Data availability

All data underlying the results are available as part of the article and no additional source data are required.

## Consent

Written informed consent for publication of clinical and laboratory details was obtained from the patient.

## References

[1] Cole LA : Familial hCG Syndrome. *J Reprod Immunol.* 2012;93(1):52–7. 10.1016/j.jri.2011.11.001 22188758

[2] Cole LA Butler S : Familial hcg syndrome: Production of variable, degraded or mutant forms of hcg. *J Reprod Med.* 2014;59(9–10):435–42. 25330684

[3] Tan A van der Merwe AM Low X : Familial HCG syndrome: A diagnostic challenge. *Gynecol Oncol Rep.* 2014;10:47–8. 10.1016/j.gynor.2014.05.005 26082938 PMC4458743

[4] Cole LA : Biological functions of hCG and hCG-related molecules. *Reprod Biol Endocrinol.* 2010;8:102. 10.1186/1477-7827-8-102 20735820 PMC2936313

[5] Talmadge K Boorstein WR Fiddes JC : The Human Genome Contains Seven Genes for the beta-Subunit of Chorionic Gonadotropin but Only One Gene for the beta-Subunit of Luteinizing Hormone. *DNA.* 1983;2(4):281–9. 10.1089/dna.1983.2.281 6319099

[6] Khattri S Vivekanandarajah A Varma S : Secretion of beta-human chorionic gonadotropin by non-small cell lung cancer: A case report. *J Med Case Rep.* 2011;5:19. 10.1186/1752-1947-5-19 21247424 PMC3036637

[7] Miller C McQuade M MacCallum J : Persistently elevated hCG in a patient with no clear evidence of pregnancy. *Clin Chim Acta.* 2020;510:703–6. 10.1016/j.cca.2020.09.004 32910977

[8] Turner JH Ross H Richmon J : Secretion of beta-HCG from squamous cell carcinomas of the head and neck. *Otolaryngol Head Neck Surg.* 2010;143(1):169–70. 10.1016/j.otohns.2010.02.036 20620641

[9] Sturgeon CM Berger P Bidart JM : Differences in recognition of the 1st WHO international reference reagents for hCG-related isoforms by diagnostic immunoassays for human chorionic gonadotropin. *Clin Chem.* 2009;55(8):1484–91. 10.1373/clinchem.2009.124578 19541864

[10] Whittington J Fantz CR Gronowski AM : The analytical specificity of human chorionic gonadotropin assays determined using WHO International Reference Reagents. *Clin Chim Acta.* 2010;411(1–2):81–5. 10.1016/j.cca.2009.10.009 19843470

[11] Cole LA DuToit S Higgins TN : Total hCG tests. *Clin Chim Acta.* 2011;412(23–24):2216–22. 10.1016/j.cca.2011.08.006 21864517

