# Supplementary material for: Case Report: The first familial hCG syndrome in a Chinese family
Source: F1000Res. 2021 Jun 8;10:458. [Version 1] doi: 10.12688/f1000research.53636.1 (PMC8240598; doi:10.12688/f1000research.53636.1)
Supplement: Peer review history

# Peer review: Reviewer response for version 1

**DOI:** 10.5256/f1000research.57046.r87100

**Published:** 2021-06-28

**Authors:** Ravi Krishna Cheemakurthi

**Affiliations:** 1 Center for Assisted Reproduction, Krishna IVF Clinic, Visakhapatnam, Andhra Pradesh, India

I appreciate authors for their effort in presenting the case report with clinical findings and proper diagnostic parameters. My suggestion is it would have been more exciting if authors would have tried to find the genetic cause behind the clinical condition, because in many rare disorders it is very difficult to find a family member with similar clinical condition. As of now it is just a case with familial hCG syndrome which was  first case in a Chinese family.

Are enough details provided of any physical examination and diagnostic tests, treatment given and outcomes?

Yes

Is the case presented with sufficient detail to be useful for other practitioners?

Yes

Is sufficient discussion included of the importance of the findings and their relevance to future understanding of disease processes, diagnosis or treatment?

Yes

Is the background of the case’s history and progression described in sufficient detail?

Yes

Reviewer Expertise:

My area of research is in the field of reproductive genetics especially in the pharmocogenomic aspects of LH and FSH supplementation in COS protocols

I confirm that I have read this submission and believe that I have an appropriate level of expertise to confirm that it is of an acceptable scientific standard.

# Peer review: Reviewer response for version 1

**DOI:** 10.5256/f1000research.57046.r87096

**Published:** 2021-06-16

**Authors:** Weldon Chiu

**Affiliations:** 1 Department of Chemical Pathology, Labplus, Auckland City Hospital, Auckland, New Zealand

A very well written and worked-up case report on this rare but clinically important condition to avoid over-investigation or worse still, potentially invasive and harmful treatments offered for a benign condition. Though there is no gold standard test, the arguments in favour of this diagnosis are scientifically sound. My only suggestion is that especially with the rarity of this condition, if there are any additional information including serial hCG results and/or any free beta HCG results e.g. from Autodelfia or Johnson and Johnson free beta-hCG platform (designed mainly for maternal serum screening), they would be worth mentioning in the report too.

Are enough details provided of any physical examination and diagnostic tests, treatment given and outcomes?

Yes

Is the case presented with sufficient detail to be useful for other practitioners?

Yes

Is sufficient discussion included of the importance of the findings and their relevance to future understanding of disease processes, diagnosis or treatment?

Yes

Is the background of the case’s history and progression described in sufficient detail?

Yes

Reviewer Expertise:

Gastrointestinal and liver related clinical biochemistry; General chemical pathology. Had encountered 2 families with familial hCG syndrome.

I confirm that I have read this submission and believe that I have an appropriate level of expertise to confirm that it is of an acceptable scientific standard.
